# Tomatoes as Food

**Published:** 1871-02

**Authors:** 


### TOMATOES AS FOOD.

It is known that the essence of the tomato made into a pill acts upon the liver, and to that extent must counteract biliousness and all forms of fever.

The free use of figs is known to multitudes to obviate constipation in a great many cases; every intelligent druggist knows that a table-spoon of white mustard-seed, swallowed without chewing, is useful in the same direction, has been used for that purpose for a century, and for that reason is kept in every good drug store for sale. The seeds pass from the body unchanged, but are supposed "to act" on the bowels mechanically. The seeds of the delightful tomato act in the same manner; hence the fruit, while it is palatable to the taste, and nutritious to the body, has a health-promoting effect on the liver and the whole digestive system. And yet a loose statement is made in some of the papers that

"Tomatoes are unhealthful. For they can cause salivation. Proof. A young lady lost all her teeth from the excessive use of tomatoes."

The writer was salivated many times in youth, and yet few persons of his age have sounder teeth and more of them.

A young girl, two years ago, in Pennsylvania, from the excessive use of "ice-cream," the fourteenth saucerful on the same evening, did not exactly lose her teeth, but she lost her life.

General Taylor, while President of the United States, lost his life by eating "one more" saucerful of strawberries and cream.

Many a man has died of a surfeit of roast beef; if an article of food so delicious, so cheap, so abundant as the tomato is to be banished from the table because once in a century, and once in a million of cases its "excessive" use killed somebody, we shall soon have nothing to eat. The newspaper press owes it to the public and its own intelligence to keep such palpably loose statements out of its columns. This slap dash kind of way, which some writers have regardless of common sense, ought to be universally discountenanced by gentlemen of the press.

